# Global systematic review and meta-analysis of health-related quality of life in Behcet’s patients

**DOI:** 10.22088/cjim.13.3.447

**Published:** 2022

**Authors:** Maryam Masoumi, Alireza Sharifi, Sepideh Rezaei, Sima Rafiei, Hossein Hosseinifard, Saghar Khani, Maryam Doustmehraban, Mona Rajabi, Zahra Beiramy Chomalou, Parisa Soori, Akbar Javan Biparva, Afsaneh Dehnad, Fatemeh Pashazadeh kan, Ahmad Ghashghaee

**Affiliations:** 1 Clinical Research and Development Center, Qom University of Medical Sciences, Qom, Iran; 2 School of Health Management and Information Sciences, Iran University of Medical Sciences, Tehran, Iran; 3 Social Determinants of Health Research Center, Research Institute for Prevention of Non-Communicable Diseases, Qazvin University of Medical Sciences, Qazvin, Iran; 4 Research Center for Evidence-Based Medicine, Tabriz University of Medical Sciences, Tabriz, Iran; 5 School of Medicine, Iran University of Medical Sciences, Tehran, Iran; 6 Student Research Committee, School of Nursing and Midwifery, Iran University of Medical Sciences, Tehran, Iran; 7 Student Research Committee, School of Health Management and Medical Informatics, Iranian Center of Excellence in Health Management, Tabriz University of Medical Sciences, Tabriz, Iran.; 8 School of Health Management and Information Sciences, Center for Educational Research in Medical Sciences (CERMS), Iran University of Medical Sciences, Tehran, Iran; 9 Student Research Committee, Qazvin University of Medical Sciences, Qazvin, Iran

**Keywords:** HRQOL, Health-related quality of life, Behcet’s disease, Old Silk Route disease

## Abstract

**Background:**

Behcet’s disease (BD) is a chronic fatal illness with a relapsing remitting nature and significant organ-threatening morbidity and mortality. The aim of this research was to examine studies which were conducted on investigation of prevalence of quality of life among patients with Behcet’s disease.

**Methods:**

A total of 13 articles were extracted from four main databases including PubMed, EMBASE, Scopus, and Web of Science from the onset of 2000 to January 2021. All studies published in English with the purpose of examining quality of life (QOL) among patients with BD or investigating its main determinants were included.

**Results:**

Totally, 1137 BD patients participated in 13 studies. Based on random effect analysis, the total score of physical health-related QOL was 46.7 (95% CI=41.26 to 52.13) and the total score of mental health-related QOL was 49.01 (95% CI=43.83 to 54.18) representing a moderate level of QOL among BD patients. Furthermore, weighted effect size analyses showed a significant correlation between QOL and variables such as patients’ age, gender, disease duration and depression (pvalue: 0.00).

**Conclusion:**

As the symptoms of BD worsen over time, patients confront with more severe body pain, mobility restrictions, and difficulties in chewing, eating, speaking and swallowing which negatively affect social interactions of patients and reduce their QOL. Furthermore, depression was proved to act as a deteriorating factor for Health-Related Quality of Life (HRQOL) among BD patients. Thus, patients need to be psychologically supported by a specialized team and be informed during the course of treatment to gain useful information about the disease, treatment approaches and coping strategies.

Behcet's disease (BD) is a rheumatologic disorder that was discovered first by a dermatologist, Hulusi Behcet in 1937 (1). The disease has an unknown etiology and recurrent pattern characterized by oral and genital ulcerations. It has also the capacity to involve almost all body organs such as gastrointestinal tract, skin, mucosa, ocular, vascular system, joints, pulmonary, urogenital, musculoskeletal, cardiac and the nervous system leading to significant morbidity and mortality. A relapsing-remitting disorder, inflammatory responses, and positive response to immunosuppressive therapy in BD are the main indications for an auto-inflammatory-autoimmune nature of the disease (2-7). BD is more common in Mediterranean, Middle Eastern and Far Eastern countries including Turkey, Greece, Iraq, Iran, Japan and China. The higher prevalence of the disease in mentioned areas might be due to certain genetic and environmental factors. Age and gender are other features that might increase the risk of Behcet’s. Generally, the disease affects people aged 20–40 years and is more severe among men (8, 9).

Common symptoms of the disease include arthritis, oral aphthae, skin lesions and ulcers in the genital area which may also develop ocular and vascular complications (3). These symptoms’ negative effects on the physical and mental health of patients, which consequently diminish their quality of life (QOL). 

It was also declared that oral ulcers might affect the body image negatively and restrain the process of nourishment and speech in affected individuals (10, 11). Patients might generally suffer from weight loss, depression and tiredness during the progressive course of the disease. On the other hand, chronic rheumatologic problems in these patients limit mobility and their daily activities leading to impaired self-esteem which negatively affects their ability to manage constructive relationships with others (3, 12). The World Health Organization (WHO) suggested a definition of Health-Related Quality of Life (HRQOL) in 1993 as a concept that includes being good at social relationships, feeling good physically and emotionally, having the ability to satisfy a person's basic needs (2), and being healthy in social, mental and psychological areas of life (13). 

Several studies have indicated the negative impact of Behcet's disease on patients’ QOL (2). In a study done by Gorial et al. in Iraq the total score of QOL in the patient group was 51.8±22 compared with the control group (91.7±2.6) (14). Additionally, Guler et al. estimated the mean score of physical and mental QOL among BD patients in Turkey at 73.43 and 57.85, respectively (15). Literature affirmed that body pain accompanied by skin lesions could deteriorate the psychological and physical well-being of BD patients and reduce their QOL through disturbances to systematic physiological functions. 

In a research conducted among Iranian population, Davatchi et al. found that the most common manifestations were genital and oral aphthosis, ocular complications and skin disorders (1). In addition, Fabiani et al. (16) and Canpolat et al. (2) showed that patients with BD had a lower QOL compared to healthy subjects. They added that measuring QOL can help to better understand the behaviors and social interactions of patients as well as difficult situations they experience while dealing with the disease complications (17). This information can be used for developing all-inclusive care plans to help patients effectively manage their illness and develop coping strategies (18).

In fact, a holistic point of view to best manage the deteriorating impacts of BD on health-related QOL demands an aggregation of a large body of research evidence containing information about the status of patients’ QOL to be considered in clinical decision making. Despite the fact that BD disease negatively affects patients socially, physically and mentally and significantly decreases their QOL, few studies have systematically reviewed HRQOL and examined its determinants among BD patients. Thus, this research was done to evaluate QOL in BD patients and evaluating the association between QOL and their socio-demographic and disease characteristics (including age, gender, disease duration, depression, etc.).

## Methods

**Registration: **This study was registered in PROSPERO at the University of York (registration code: CRD CRD42021225497; available at https://www.crd.york.ac.uk/prospero/display_record.php?ID=CRD42021225497). 

**Databases and search terms: **The systematic literature search was performed in electronic databases of PubMed, Web of Science, Scopus, CINAHL, EMBASE and Google Scholar between the onset of 2000 and January 2021 through the search terms including ((life quality [Title/Abstract]) OR (Health Related Quality of Life [Title/Abstract]) OR (HRQOL[Title/Abstract]) AND (Behcet's Disease[Title/Abstract]) OR Behcet’s Disease[Title/Abstract]) OR (Behcet's Syndrome[Title/Abstract]) OR (Triple Symptom Complex[Title/Abstract]) OR (Behçet’s Disease[Title/Abstract]) OR (Behçet’s Diseases[Title/Abstract]) OR (Adamantiades Behcet’s Disease[Title/Abstract]) OR (old Silk Route Disease[Title/Abstract])). 

**Selection process: **Through an initial search of the literature, 521 articles were found. Then the records were entered to EndNote software and 180 duplicates were removed. In the next step, title/ abstracts of 341 remaining articles were screened by two research members to exclude irrelevant records; of which 97 articles were published in PubMed, 147 in SCOPUS, 42 in Web of Science and 55 articles were retrieved from EMBASE. 

**Figure 1:**
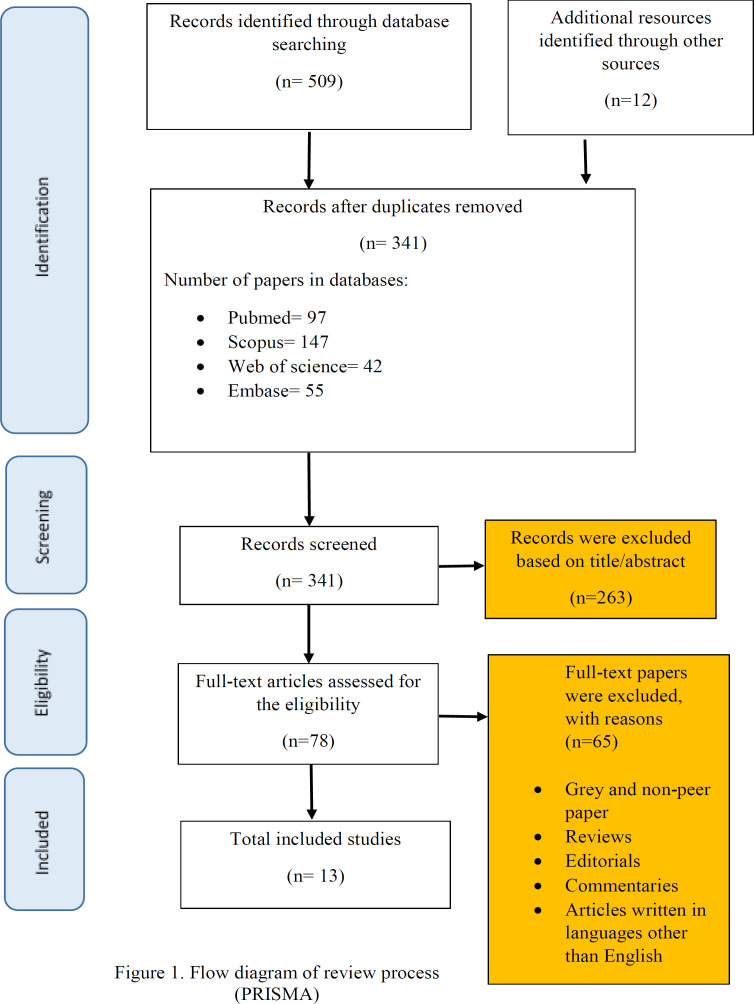
Flow diagram of review process (PRISMA)

The screening process led to 78 relevant records which remained to be assessed for the eligibility. After considering inclusion/ exclusion criteria, 13 articles remained (figure 1).

**Exclusion and Inclusion criteria: **The inclusion criteria were original research papers with available full-text, and observational designs of cross-sectional, cohort, case-series, descriptive, and prospective studies published in English from the beginning of 2000 to January 2021 to measure the health-related quality of life among BD patients or identify its main determinants. Papers in languages other than English, published before January 2000 or after January 2021 were excluded from the review. Furthermore, review articles, letter to the editor, commentaries, expert opinions, case studies, case-control, books, book chapters, brief reports, randomized controlled trials and thesis were not included in the research.

Regarding the study objectives, papers addressing the topics of treatment, follow-up, medication approaches, and clinical decision-makings were also excluded from the review. 

**Data extraction: **Two investigators extracted a study data using a preliminary data extraction form encompassing general information about the included paper such as name of author/ authors, year of publication, study design, research setting, sample size, data collection tool and obtained results in terms of total score of QOL among BD patients, their demographic characteristics, duration of disease and level of depression (table 1). 

**Table 1:** characteristics of included studies

First Author	Year of Publication	Country	Continent	WHO regions	Total	Total Number of Male	Total Number of Female	Age	Duration of disease (Years)	Depression	Tools	References
Atas,et al	2019	Turkey	Europe	EURO^*^	55	43	12	36.8	4.1	23.14	SF-36	(19)
Buyuktas,et al	2015	Turkey	Europe	EURO	152	73	79	37.5	6.2	15.36	SF-36	(20)
Canpolat,et al	2011	Turkey	Europe	EURO	94	47	47	45.6	9.5	18.26	SF-36	(2)
Eren,et al	2006	Turkey	Europe	EURO	54	30	24	39.35	9.07	11.16	SF-36	(21)
Ertam.et al	2009	Turkey	Europe	EURO	195	102	93	38.77	10.5	20.14	SF-36	(22)
Fabiani,et al	2017	Italy	Europe	EURO	37	17	20	46.2	13.3	16.74	SF-36	(16)
Güler,et al	2017	Turkey	Europe	EURO	67	39	28	38.18	10.6	14.1	SF-36	(23)
Ilhan,et al	2016	Turkey	Europe	EURO	123	52	71	39.4	7.8	15.9	SF-36	(24)
Gorial,et al	2020	Iraq	Asia	EMRO^**^	71	45	26	36.0	5.1	21.78	SF-36	(9)
Garip, et al	2015	Turkey	Europe	EURO	50	30	20	36.7	8.5	14.69	SF-36	(25)
Toprak, et al	2016	Turkey	Europe	EURO	97	42	55	45.26	6	18.3	SF-36	(26)
Kumcu, et al	2020	Turkey	Europe	EURO	40	20	20	44.35	7.5	25.36	SF-36	(12)
Melikoğlu, et al	2014	Turkey	Europe	EURO	102	54	48	36.25	7.25	17.64	SF-36	(27)

*European Region Office

** Eastern Mediterranean Region Office

**Quality assessment: **The quality of included articles was evaluated by Newcastle-Ottawa Scale (NOS). The quality of studies were assessed by two independent reviewers to reduce possible bias. In case of disagreement between the evaluators, the article was discussed in the presence of a third observer until a consensus was reached. In the scoring system, a maximum of 9 points could be given to each of the articles; those having a total score of ≥7 were defined as high quality and those with a score below 4 were mentioned as low quality ones (28).

**Statistical Analysis: **We used the Der Simonian-Laird model and validated our study results using sensitivity analysis. According to the sample size and publication year, the statistical heterogeneity was assessed using the I^2 ^statistics. For reducing the effect of heterogeneity, a subgroup analysis was done in the areas of study design, study setting, the research quality, sample size, and publication year. After applying the Egger test for assessing the publication bias, Comprehensive Meta-Analysis software was exploited to analyze data. 

## Results

**Figure 2:**
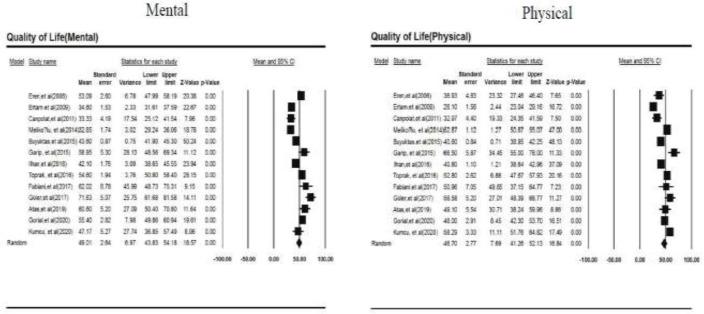
Forest plots of both physical and mental health-related quality of life (HRQOL)

Our findings are reported based on the PRISMA checklist (Preferred Reporting Items for Systematic Reviews and Meta-Analyses) (29). Following the extraction of data from 13 articles, the total number of patients was 1137. Based on the study results, the total physical and mental HRQOL in patients with BD was calculated at 46.70±2.77 (95% CI 41.26 to 52.13) and 49.01±2.64 (95% CI 43.83 to 54.18), respectively (figure 2).

**Figure 3:**
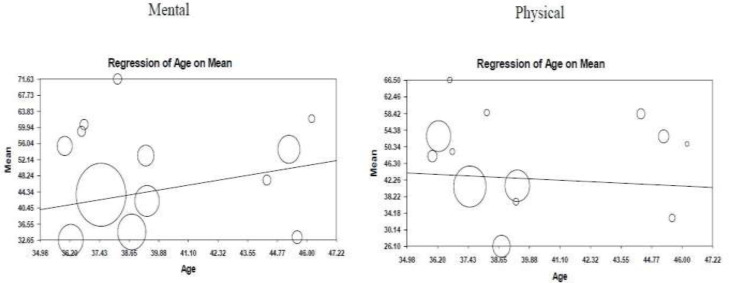
Meta-regression based on age

**Meta regression for age: **The results indicated a significant reverse correlation between physical HRQOL and age. It means that, a unit of increase in patients’ age decreased their quality of life by 0.28. On the other hand, positive direction of association between mental QOL and age was confirmed so that there was a unit of increase in patients’ age increased their mental health by 0.97(figure 3).

**Figure 4:**
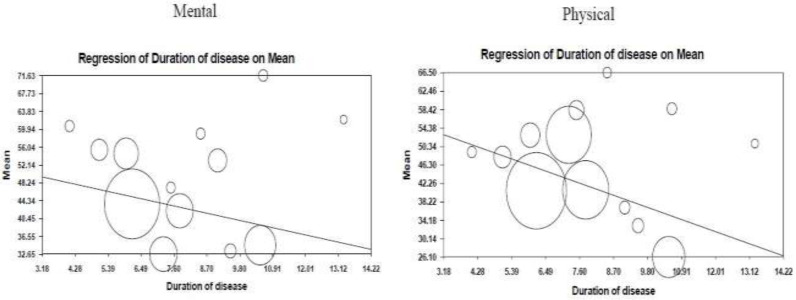
Meta-regression based on duration of disease

**Meta regression for disease duration: **The meta-regression results also illustrated that mental and physical HRQOL in patients with BD were associated with length of disease; so that a year of increase in the disease duration reduced the score of physical and mental QOL by 2.4 and 1.43, respectively (figure 4).

**Figure 5:**
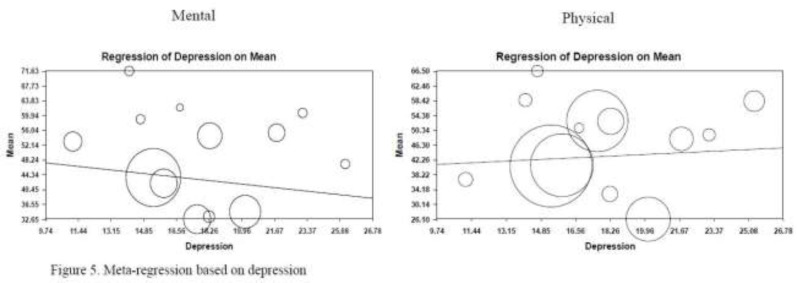
Meta-regression based on depression

**Other sub group Depression: **Study results showed a significant inverse correlation between depression and mental QOL; indicating that mental HRQOL decreased by 0.54 for each unit of increase in depression score. Conversely, depression was directly associated with physical QOL; confirming an increase by 0.26 for each unit of increase in depression (figure 5).

**Figure 6:**
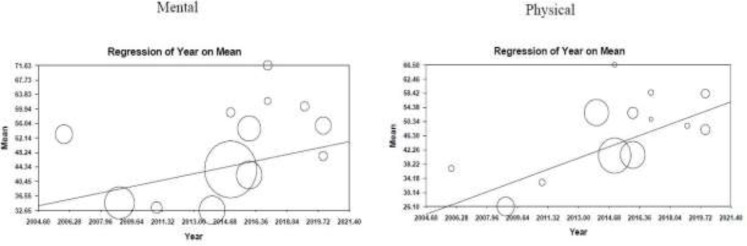
Meta-regression based on year

**Meta-Regression based on Year of Publication: **Based on the analysis, there was a significant correlation between patients’ physical HRQOL and the year of publication. In fact for every one-year increase in the publication date, physical QOL increased by 1.9 while mental QOL decreased by 1.029 (figure 6).

**Figure 7:**
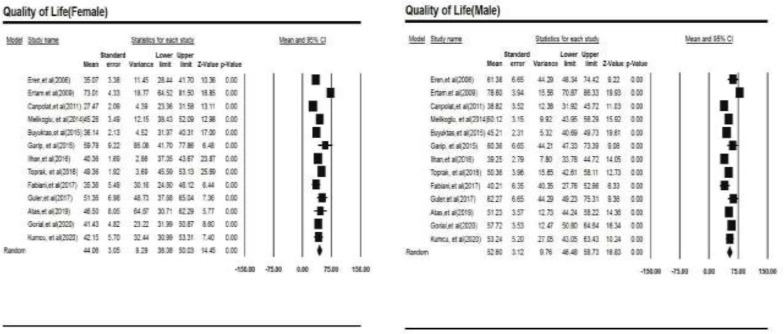
Meta-regression based on gender

**Meta regression for gender: **The total score of HRQOL in female and male patients was 44.06±3.05 (95% CI 38.08 to 50.03) and 52.60±3.12 (95% CI46.48 to 58.73), respectively. Furthermore, there was a unit of increase in females’ age increased their quality of life by 0.59 while there was a unit of increase in males’ age decreased their quality of life by 0.69 (figure 7). 

**Meta regression based on SF-36 questionnaire items: **Data were analyzed based on SF-36, according to which the total score of each item was: physical functioning: 68.71 (95% Cl, 64.28-73.13), social functioning: 68.82 (95% Cl, 65.39-72.24), role physical: 52.10 (95% Cl, 44.83-59.37), role emotional: 53.51(95% Cl, 43.71-63.30), mental health: 57.29(95% Cl, 54.37-60.21), vitality: 49.34 (95% Cl, 43.93-54.75), body pain 55.46(95% Cl, 50.95-59.96), general health 47.70(95% Cl, 43.62-51.78), physical component summary 46.16 (95% Cl, 40.84-51.49), and mental component summary 49.46(95% Cl, 42.82-56.11) (table 2).

**Table 2:** Meta regression based on SF-36 questionnaire items

** Subsections **	**Mean**	**Lower limit**	**Upper limit**	**P-value**
Physical functioning	68.71	64.28	73.13	0.00
Social functioning	68.82	65.39	72.24	0.00
Role–physical	52.10	44.83	59.37	0.00
Role–emotional	53.51	43.71	63.30	0.00
Mental health	57.29	54.37	60.21	0.00
Vitality	49.34	43.93	54.75	0.00
Bodily pain	55.46	50.95	59.96	0.00
General health	47.70	43.62	51.78	0.00
Physical Component Summary (PCS)	46.16	40.84	51.49	0.00
Mental Component Summary (MCS)	49.46	42.82	56.11	0.00

## Discussion

This is the first systematic review and meta-analysis which has been conducted in the last decade to assess QOL among BD patients and examine its determining factors. The results of SF-36 items revealed that mental and physical QOL among BD patients was at 46.7 and 49.01, respectively. Based on the literature, there is no acceptable range of scores to evaluate QOL by SF-36. Therefore, we categorized patients’ QOL into five sections through the information obtained from the expert interviews. These classifications were ‘Very poor QOL (0 to 20)’, ‘Poor QOL (21 to 45)’, ‘Moderate QOL (46 to 55)’, ‘Good QOL (56 to 65)’ and ‘Very good QOL (66 to 100)’. Generally, the closer to 100 indicates a better HRQOL (30). Furthermore, based on the estimated HRQOL in this study, both the mental and physical qualities of life scores among BD patients were evaluated in a moderate level. Similarly previous studies affirmed the deteriorating effect of Behçet’s disease on patients’ QOL and found that BD patients had lower scores of QOL (31-34). Experiencing high levels of pain and discomfort as well as severe difficulties in everyday activities were considered as the most important reasons for poorer quality of life among adults with BD (32). These discomforting conditions negatively influence the scores of HRQOL particularly regarding the role-physical, physical functioning, vitality, role-emotional, general health and bodily pain (2).

Our review revealed that age was directly associated with patients’ QOL in terms of mental health aspects while it was shown to have a significant reverse relationship with physical domains of HRQOL. In a study conducted by Canpolat and Yurtsever in 2011 findings affirmed that patients between 31 and 41 years of age had lower mean scores of QOL particularly on social functioning and general health as they are overwhelmed with a variety of tasks and responsibilities from household duties to work-related and social time activities (35). Thus, these patients have to confront the limitations imposed by their disease symptoms while doing their duties at home and the workplace. On the other hand, an improving trend in the mental HRQOL of older persons might be due to the fact that these individuals have learned how to cope with frequent symptoms during the course of their disease which consequently lead to less discomfort with pain recurrences (2). While an inverse relationship between age and physical HRQOL has suggested that pain management in older adults is much more difficult which further slows down their movement and brings numerous challenges for them. Furthermore, as the symptoms of BD worsen over time, functional capacity and physical ability of patients decrease with age and cause performance restrictions in daily activities. 

In our study, we found that female patients had a poorer QOL on the physical health domain in comparison with males. This finding agrees with most of the literatures depicting that women have poorer health-related quality of life than men (36). This variation might be due to the physiological and hormonal differences between men and women or might be attributable to some of the gender-role beliefs mentioning women as a weaker sex who are less capable than men in accomplishing physical and social activities. Peacock and Weston added an explanation for poorer HRQOL among women. The implication was that female patients did not only have chronic pain and physical illness due to long-term family care giving roles but also suffered from role pressures associated with work and family domains (36). 

In our review, depression was mentioned as a deteriorating factor for HRQOL among BD patients; which in some cases paved the way for typical signs and symptoms of BD.(37, 38) Literature also highlighted the consistent association between BD and depression, with an incidence of 86% at onset of disease symptoms and disorders (depression as). Furthermore, in several studies depression, anxiety and fatigue were mentioned as factors influencing QOL in BD patients (39, 40). In a study conducted by Fawzy et al. 74.3% of BD patients revealed depressive symptoms, while a similar research in Korea and Turkey reported the frequency rate to be 46% and 40.6% respectively (34, 41). Similarly in a study conducted by Dursun et al., findings revealed that approximately 1/5 of BD patients were depressed (42). Thus, BD patients who have both physical and mental symptoms need early preventive programs and depression management interventions to reduce mental disorders and improve their QOL. Due to the complex multifaceted nature of disease BD patients need a comprehensive treatment approach in which motivational, and behavioral strategies are used to assist them in successfully changing their health-related behaviors toward improving QOL. Guler et al. and Fabiani et al. agreed with our study findings and mentioned depression as a confounding factor for QOL evaluations (3, 4, 43, 44). They also emphasized on the necessity for depression screening and follow-up as a routine clinical assessment of BD patients. Several mechanisms were also suggested to explain these interactions. First, it is proposed that the pro-inflammatory cytokines in the pathogenesis of Behcet's disease cause acute flares that may have neural effects associated with depressive symptoms (45). Second, functional disability may contribute to subsequent depressive symptoms by restricting physical mobility and daily activities (42). 

Also we found duration affected QOL scores. In line with our findings, literature confirmed that the patients’ social health declined over the course of the disease (4, 16). In a study by Canpolat and Yurtsever, 23.4% of patients were adversely affected by the disease symptoms and their role-emotional, social functioning, and general health decreased considerably (2). As the symptoms of BD worsen over time, patients confront with more severe body pain, mobility restrictions, and difficulties in chewing, eating, speaking and swallowing which negatively affect social interactions of BD patients and reduce their QOL. Thus, patients need to be psychologically supported by a specialized team and be informed periodically during the course of treatment to gain useful information about the disease, treatment approaches and coping strategies. 

Mumcu et al. found that physical symptoms of Behçet’s disease negatively affected QOL subscales. In their study, BD patients were reported to have lower mean scores on physical functioning, role-physical, body pain and general health compared to healthy adults (46). Similarly, Bodur et al. found that mean scores on psychosocial and physical domains were lower for BD patients (33). 

The prim limitation of this study is that we only included the studies published in English which might lead to a language bias in the research. Second, we did not assess the associations between involvement of different organ systems and quality of life. Third, we only included studies which used SF-36 to assess HRQOL among the BD patients. Fourth, mental disorders other than depression were not included in the current research which limited our findings.  

In our review, the physical and mental quality of life among the BD patients was evaluated in a moderate level. We found that patient’s demographic features such as age and gender and disease-related factors including disease duration and depressive symptoms affected QOL scores. Depression was proved to act as a deteriorating factor for HRQOL among BD patients. Thus, patients need to be psychologically supported by a specialized team and be informed periodically during the course of treatment to gain useful information about the disease, treatment approaches and coping strategies. The disease management interventions should also take a gender-based perspective that provides special supporting programs for women who have poorer physical health-related quality. Furthermore, as the symptoms of BD worsen over time, patients confront with more severe body pain, mobility restrictions, and difficulties in chewing, eating, speaking and swallowing which negatively affect social interactions of patients and reduce their QOL. These results highlight the importance of disease management strategies to cope effectively with the disease symptoms.
